# FGF21 resistance is not mediated by downregulation of beta-klotho expression in white adipose tissue

**DOI:** 10.1016/j.molmet.2017.03.009

**Published:** 2017-03-27

**Authors:** Kathleen R. Markan, Meghan C. Naber, Sarah M. Small, Lila Peltekian, Rachel L. Kessler, Matthew J. Potthoff

**Affiliations:** 1 Department of Pharmacology, University of Iowa Carver College of Medicine, Iowa City, IA 52242, USA; 2 Fraternal Order of Eagles Diabetes Research Center, University of Iowa Carver College of Medicine, Iowa City, IA 52242, USA

**Keywords:** FGF21, Resistance, Betaklotho, Obesity, Adipose

## Abstract

**Objective:**

Fibroblast growth factor 21 (FGF21) is an endocrine hormone that regulates metabolic homeostasis. Previous work has suggested that impairment of FGF21 signaling in adipose tissue may occur through downregulation of the obligate FGF21 co-receptor, β-klotho, which leads to “FGF21 resistance” during the onset of diet-induced obesity. Here, we sought to determine whether maintenance of β-klotho expression in adipose tissue prevents FGF21 resistance and whether other mechanisms also contribute to FGF21 resistance in vivo.

**Methods:**

We generated adipose-specific β-klotho transgenic mice to determine whether maintenance of β-klotho expression in adipose tissue prevents FGF21 resistance in vivo.

**Results:**

β-klotho protein levels are markedly decreased in white adipose tissue, but not liver or brown adipose tissue, during diet-induced obesity. Maintenance of β-klotho protein expression in adipose tissue does not alleviate impaired FGF21 signaling in white adipose or increase FGF21 sensitivity in vivo.

**Conclusions:**

In white adipose tissue, downregulation of β-klotho expression is not the major mechanism contributing to impaired FGF21 signaling in white adipose tissue.

## 1 Introduction

Fibroblast growth factor 21 (FGF21) is a member of the FGF19 subfamily of fibroblast growth factors (FGFs) and is an important regulator of nutrient and energy homeostasis. Pharmacological administration of FGF21 to obese and diabetic animal models has significant therapeutic effects including improving insulin sensitivity and decreasing body weight (reviewed in [1]). In humans, FGF21 analogs also reduce body weight and improve metabolic profiles in obese and diabetic patients [2], [3]. In contrast to its pharmacological actions, circulating levels of FGF21 are regulated physiologically by various nutritional cues and cellular stress. For example, plasma FGF21 levels are elevated in both rodents and humans in response to high carbohydrate levels [4], [5], [6], protein restriction [7], [8], fasting [9], [10], [11], [12], [13], [14], and obesity and insulin resistance [14], [15], [16], [17], [18], [19], [20], [21], [22].

FGF21 signals to target cells through a receptor complex consisting of the FGF receptor (FGFR), FGFR1c, and a co-receptor termed β-klotho [23], [24]. Although FGFR1c is ubiquitously expressed, β-klotho expression is restricted to specific metabolic tissues including adipose tissues, liver, pancreas, and specific regions of the brain [25], [26] and confers specificity for FGF21 signaling. Activation of the FGF21 receptor complex initially activates phosphorylation of FGF receptor substrate 2α (FRS2α) and the MAPK signaling cascade resulting in ERK1/2 phosphorylation [23], [24], [27]. Multiple studies have implicated adipose tissues in mediating the physiological and pharmacological effects of FGF21 on metabolism. A role for FGF21 in regulating metabolism was originally identified in a screen for factors that induce glucose uptake in white adipocytes [28]. Subsequent studies found that FGF21 markedly improves insulin sensitivity in rodents both acutely and following chronic administration [29], [30], [31], and adipose tissues were implicated in mediating these metabolic effects. For example, elimination of either FGFR1 [32] or β-klotho [33] from adipose tissues impairs the acute insulin sensitizing effects of FGF21. In addition, the metabolic effects of FGF21 [34] and FGFR1-agonists (activating antibodies) [35] in lowering triglyceride and glucose levels are markedly impaired in lipodystrophic mice. Transplantation of white adipose tissue from wild-type mice into lipodystrophic mice subsequently restored FGF21 responsiveness [34]. Finally, increases in adipose tissue ‘browning’ [30], [36] and brown adipose tissue (BAT) glucose and triglyceride uptake [33], [37], [38] have been observed in response to FGF21 administration.

Pharmacological administration of FGF21 has pronounced metabolic effects even though circulating endogenous levels of FGF21 are markedly elevated in obese rodents [15], [16], [17], [18], [19], monkeys [39], and humans [20], [21], [22]. The elevated FGF21 levels during obesity have led to the postulation that obesity is a “FGF21-resistant state” [17]. However, the concept of FGF21 resistance is controversial as a subsequent study demonstrated that FGF21 sensitivity is not impaired in DIO WT and *ob/ob* mice compared to lean controls [18]. Both studies identified a reduction in β-klotho expression in adipose tissue of obese rodents [17], [18], an effect also observed in white adipose tissue of obese humans [40]. Thus, downregulation of β-klotho expression in adipose tissue could impair endogenous FGF21 action and contribute to FGF21 resistance. Here we show that while β-klotho expression in white adipose tissue is markedly reduced during diet-induced obesity, maintenance of β-klotho expression in adipose tissue, through the generation of adipose-specific β-klotho transgenic mice, does not increase FGF21 sensitivity or significantly improve metabolic parameters during obesity. We propose that selective downregulation of β-klotho expression in white adipose tissue may function as a beneficial adaptation, rather than a pathological impairment, in FGF21 action to regulate energy homeostasis.

## 2 Materials and methods

### 2.1 Animals

Adiponectin-cre transgenic mice have been reported previously [41]. To generate inducible KLB transgenic (TG) mice, a full-length mouse β-klotho cDNA clone was purchased (Thermo Scientific, Inc.) and cloned into the MSP universal transgenic construct [42] (kindly provided by Dr. Curt Sigmund, Univ. of Iowa). The β-klotho-MSP-Universal transgenic construct was then linearized via SphI and XhoI restriction sites and sent to the University of Iowa Genome Editing Core for injection and founder generation. To achieve adipose-specific β-klotho expression, different lines of inducible KLB TG mice were crossed with Adiponectin-Cre mice (JAX Labs) to generate KLB AdipoTG mice. Three lines of KLB AdipoTG mice were generated and screened for transgene expression levels. Line 2 was selected and then backcrossed four generations to C57Bl/6 mice before crossing to Adiponectin-cre mice for the reported studies. All mice used in these studies were male mice that were maintained on either standard chow (2920X; Envigo) or 60% high fat diet (HFD; Research Diets, D12492i) for the indicated time. To induce obesity, mice were placed on HFD starting at 4–6 weeks of age. All experiments were approved by the University of Iowa IACUC.

### 2.2 Glucose and insulin tolerance tests

Body composition was measured using a rodent-sized NMR machine (Bruker Minispec LF50) prior to glucose tolerance tests (GTTs) for determination of lean mass. Following an overnight (16 h) fast, time 0 blood was collected via tail bleed followed by an intraperitoneal (i.p.) injection of glucose (2 g glucose/kg lean body weight). Tail blood was then collected into 300K2E microvette EDTA tubes (Sarstedt) over the course of 120 min and then centrifuged at 3000 rpm for 30 min at 4 °C for the separation of plasma. Plasma glucose was then measured using the Autokit Glucose Reagent (WAKO) per manufacturer's instructions. For insulin tolerance tests (ITTs), mice were fasted 5–6 h. Time 0 blood was obtained via tail bleed followed by an intraperitoneal (i.p.) injection of insulin (at the indicated dose). Tail blood was then collected and plasma glucose analyzed as described above.

### 2.3 In vivo FGF21 signaling

Food was removed 2 h prior to the start of these experiments. Each mouse received an intraperitoneal injection of vehicle or recombinant human FGF21 (0.1 mg/kg total body weight). Fifteen minutes post-injection, mice were euthanized by decapitation for tissue collection. All tissues were snap frozen in liquid nitrogen and then stored at −80 °C until analysis.

### 2.4 Gene expression and protein analysis

Gene expression analysis was performed as described [4]. QPCR primer sequences are as follows: *Klb*: 5′-GATGAAGAATTTCCTAAACCAGGTT-3′, 5-AACCAAACACGCGGATTTC-3′; *Fgfr1c*, 5′-GCCAGACAACTTGCCGTATG-3′, 5′-ATTTCCTTGTCGGTGGTATTAACT-3′; *Tdtomato*, 5′-CGAGGAGGTCATCAAAGAGTTC-3′, 5′-GGGAAGGACAGCTTCTTGTAAT-3′; *U36b4*, 5′-CGTCCTCGTTGGAGTGACA-3′, 5′-CGGTGCGTCAGGGATTG-3′; *Fasn*: 5′-GCTGCGGAAACTTCAGGAAAT-3′, 5′-AGAGACGTGTCACTCCTGGACTT-3′; *Fgf21*: 5′-CCTCTAGGTTTCTTTGCCAACAG-3′, 5′-AAGCTGCAGGCCTCAGGAT-3′; *Pck1*: 5′-CACCATCACCTCCTGGAAGA-3′, 5′-GGGTGCAGAATCTCGAGTTG-3′; *Hmgcr*: 5′-CTTGTGGAATGCCTTGTGATTG-3′, 5′-AGCCGAAGCAGCACATGAT-3′; *Srebf1*: 5′-GGAGCCATGGATTGCACATT-3′, 5′-GGCCCGGGAAGTCACTGT-3′; *Scd1*: 5′-TGCCCCTGCGGATCTT-3′, 5′-GCCCATTCGTACACGTCATT-3′.

For protein analysis, tissues were homogenized on ice in lysis buffer containing 10 mM Tris–HCl, pH 7.4, 5 mM EDTA, 5 mM EGTA, 150 mM NaCl, 10% glycerol, 1% NP-40, 0.5% Triton X-100, and protease inhibitors. Samples were centrifuged for 5 min at 0.5×*g* at 4 °C and infranatant collected. An appropriate volume of 6X Laemmli buffer was added and all samples incubated at 100 °C for 10 min and then briefly placed on ice. Sample protein concentration was determined by Bradford assay and then equal quantity of sample resolved by SDS-PAGE. Proteins were transferred to a PVDF membrane before being probed with the specified antibodies. Antibody information: β-klotho (R&D Systems, #AF2619), β-actin (Sigma, #A5316), IR-β (Santa Cruz, #sc-711), phospho-FRS2α (Cell Signaling, #3864), phospho-ERK1/2 (Cell Signaling, #9101), total ERK1/2 (Cell Signaling, #9102), phospho-MEK (Cell Signaling, #9121), and total MEK (Cell Signaling, #9122).

For adipose tissue fractionation data, white adipose tissue was harvested from the indicated mice and processed using the Minute Adipose Tissue Fractionation Kit (Invent Biotechnologies, #AF-023) per the manufacturer's instructions.

### 2.5 Plasma and tissue analysis

Mouse FGF21 (Biovendor) and mouse insulin (Crystal Chem) were measured using commercially available ELISAs. Blood was collected into 300K2E microvettes (Sarstedt) and spun at 3,000 rpm for 30 min 4 °C to separate plasma. Plasma glucose levels were measured using the glucose autokit (Wako Chemicals). Plasma triglycerides and cholesterol were measured using colorimetric assays (Infinity™, Thermo Scientific). All measurements were performed according to the manufacturer's instructions.

Hepatic triglycerides were quantified via Folch extraction. Mouse livers were collected, snap-frozen, pulverized, and stored at −80 °C prior to analysis. Pulverized liver tissue was thoroughly homogenized for 30 s per sample in 4 ml of a 2:1 v/v chloroform/methanol mix then allowed to equilibrate at room temperature for 30 min. After adding 1 ml of 50 mM NaCl to each sample, the samples were vortexed for 15 s and centrifuged for 10 min at 1,000×*g* at room temperature. The organic phase was isolated, and 1 ml of 0.36 M CaCl_2_/Methanol/H_2_O mix (1:1 v/v Methanol/H_2_O) was added to the samples, vortexed, and centrifuged as before. The organic layer was isolated and placed into 5 ml glass volumetric flasks. The flasks were then volumed up to the 5 ml mark with fresh chloroform, capped, and left overnight at room temperature. The following day, any traces of water were carefully aspirated from the samples. In new test tubes, 10 μl of a 1:1 v/v Triton-X 100/chloroform solution was added followed by 10 μl sample. Blanks consisted of 10 μl of Triton-X 100/chloroform and 10 μl of pure chloroform. 10 μl of pre-determined standards (Verichem Laboratories Inc., Matrix Plus Chemistry Reference Kit, Cat. No 9500) in 10 μl of Triton-X 100/chloroform tubes were also included. Samples were allowed to air dry in a chemical hood overnight. The following day, hepatic triglycerides were determined using a colorimetric assay, (Infinity™, Thermo Scientific) following the manufacturer's instructions.

### 2.6 Data analysis

Statistical difference between two groups was determined via Student's t-test.

## 3 Results

### 3.1 β-klotho expression in white adipose tissue is reduced during obesity

**Figure 1:**
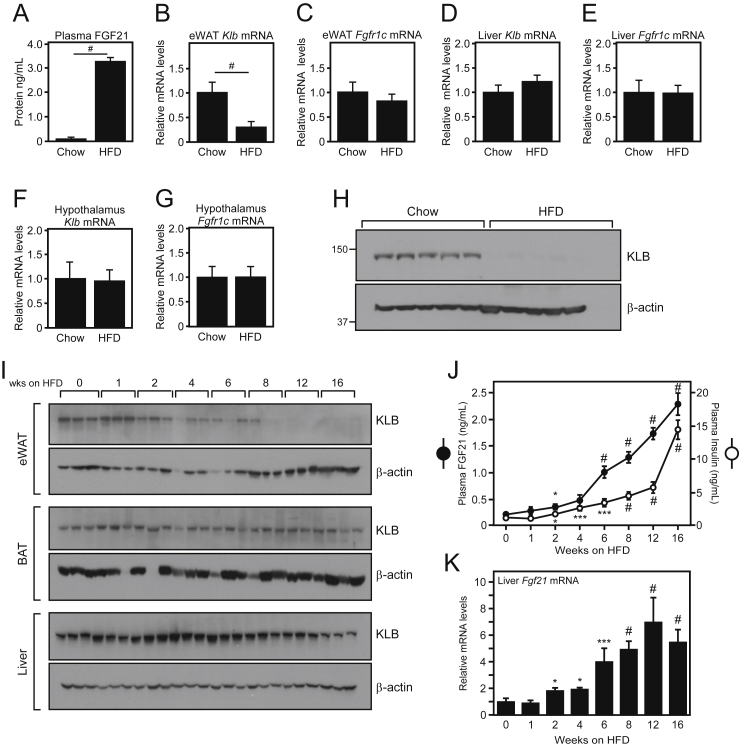
**Decreased expression of β-klotho in white adipose tissue correlates with elevated levels of circulating FGF21**. (A) Plasma FGF21 protein levels in male WT C57Bl/6 mice *ad libitum* fed chow or 60% high fat diet (HFD) for 16 weeks (n = 6–7/group). (B–G) Quantitative real-time PCR (QPCR) analysis of *Klb* and *Fgfr1c* mRNA expression in epididymal white adipose tissue (eWAT; B, C), liver (D–E), and hypothalamus (F–G) (n = 6–7/group). (H) Western blot analysis of KLB protein expression in eWAT of male WT C57Bl/6 mice *ad libitum* fed chow or 60% high fat diet (HFD) for 16 weeks. (I) Representative western blots of KLB protein expression in eWAT, brown adipose tissue (BAT), and liver of WT C57Bl/6 mice *ad libitum* fed 60% high fat diet (HFD) for the indicated time (5–6 mice/group). (J) Plasma FGF21 and insulin levels from the mice in (I). (K) Liver *Fgf21* mRNA levels from mice in (I). Values are mean +/− SEM. (*, *P* < 0.05; **, *P* < 0.01; ***, *P* < 0.005; and #, P < 0.001 compared to WT; for (J) significance is relative to time 0).

To identify potential mechanisms underlying FGF21 resistance in vivo, we compared the expression of β-klotho and FGFR1c in multiple tissues of wild-type (WT) diet-induced obese (DIO) mice, which exhibit markedly elevated circulating levels of FGF21 (Figure 1A), to that of WT lean mice. Consistent with previous publications [17], [18], *β-klotho* (gene symbol: *Klb*) mRNA levels were significantly decreased in epididymal white adipose tissue (eWAT) of DIO mice compared to lean control mice (Figure 1B). This decrease in *Klb* mRNA was not observed in other tissues including the liver and hypothalamus (Figure 1D,F). In addition, this decrease was specific for *Klb* and not observed for the FGF21 receptor *Fgfr1c* (Figure 1C,E,G). While *Klb* mRNA was significantly decreased by the HFD, KLB protein was nearly undetectable in eWAT of DIO mice (Figure 1H).

We next performed a high fat diet (HFD) feeding time course to identify when eWAT KLB protein levels decrease in relation to increased plasma FGF21 levels during the development of obesity. KLB protein levels in eWAT declined by 2 weeks, were significantly decreased by 4 weeks, and were completely absent by 12 weeks on HFD (Figure 1I). This decrease in KLB protein levels corresponded with a significant increase in circulating plasma FGF21 and insulin levels and a parallel increase in liver *Fgf21* mRNA (Figure 1J,K). In contrast to eWAT, KLB protein levels in BAT and liver were not markedly reduced (Figure 1I). Collectively, these data suggest that decreased KLB expression in white adipose tissue of DIO mice could contribute to the development of FGF21 resistance.

### 3.2 Generation of adipose-specific KLB transgenic mice

**Figure 2:**
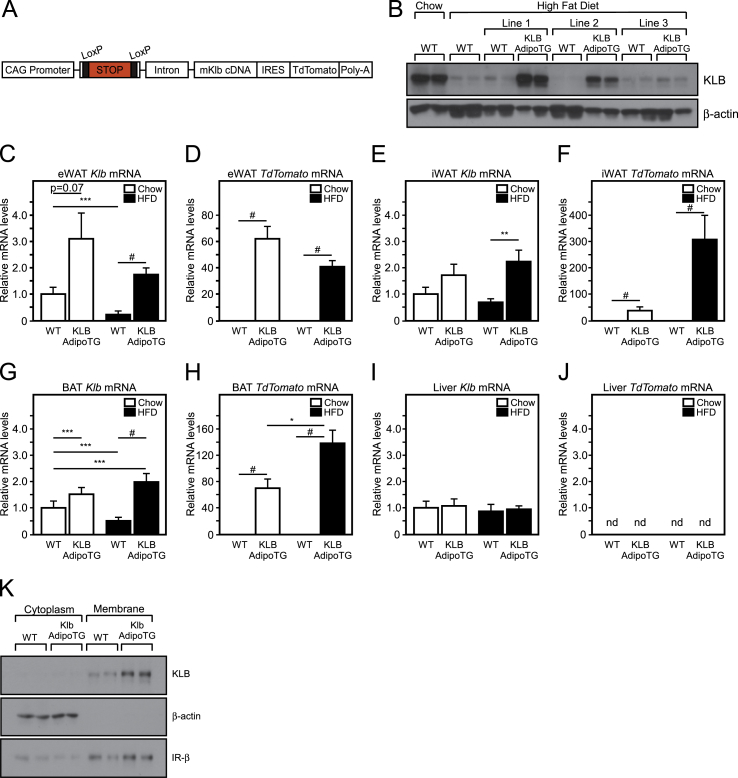
**Generation of adipose-specific β-klotho transgenic mice**. (A) Schematic of inducible β-klotho transgenic construct (inKLB). (B) Western blot analysis of KLB protein expression in epididymal white adipose tissue (eWAT) of 16 week chow-fed and high fat diet (HFD)-fed wild-type (WT) mice compared to HFD fed WT and adipose-specific β-klotho transgenic (KLB AdipoTG) mice. QPCR analysis of *Klb* and *Tdtomato* mRNA expression in eWAT (C, D), inguinal white adipose tissue (iWAT; E, F), brown adipose tissue (BAT; G, H), and liver (I, J) of WT and KLB AdipoTG mice fed chow or 60% HFD for 12 weeks (n = 7–11/group). (K) Western blot analysis of cytoplasmic and membrane KLB protein expression in epididymal white adipose tissue of wild-type (WT) and KLB adipose-specific transgenic mice (KLB AdipoTG). Values are mean +/− SEM. (*, *P* < 0.05; ***, *P* < 0.005; and #, P < 0.001 compared to WT; nd = not detected).

Based on our profiling data, we hypothesized that maintaining adipose KLB expression during diet-induced obesity would sensitize mice to endogenous, circulating FGF21, thus preventing FGF21 resistance and potentially improving insulin sensitivity. To test this, we developed adipose-specific KLB transgenic mice (KLB AdipoTG) by crossing inducible KLB transgenic mice (inKLB TG) with mice that drive Cre expression specifically in adipose tissue (Adiponectin-Cre). inKLB TG mice were generated by engineering a transgenic construct that contained a universal CAG promoter, followed by a Lox-STOP-Lox cassette, a splicable intron, then the mouse β-klotho cDNA and an IRES Tdtomato cassette followed by a poly A tail. In the absence of Cre-recombinase, the STOP sequence prevents KLB expression. When crossed to mice expressing Cre in a particular tissue, the STOP codon is removed and KLB and TdTomato is expressed in that tissue (Figure 2A). inKLB TG mice were crossed with Adiponectin-Cre transgenic mice producing KLB AdipoTG mice. Three independent lines of KLB AdipoTG mice were placed on HFD and then tested for transgene expression relative to chow and HFD fed WT mice. While HFD feeding markedly decreased eWAT KLB protein levels in WT mice, lines 1 and 2 of KLB AdipoTG mice maintained adipose tissue KLB expression on HFD (Figure 2B). Importantly, to test whether maintenance, as opposed to the supraphysiologic overexpression, of KLB levels prevents FGF21 resistance, we chose Line 2 for use in all future studies because of comparable KLB protein expression relative to chow fed WT mice (Figure 2B).

To determine the specificity for β-klotho induction in adipose tissues, we profiled *Klb* and *TdTomato* expression in lean and DIO KLB AdipoTG mice. As expected, increased *Klb* and *TdTomato* mRNA levels were only detected in adipose depots of KLB AdipoTG mice (Figure 2C–J), and KLB protein was increased in the membrane fraction of adipose tissue of KLB AdipoTG mice (Figure 2K). Under HFD conditions, *Klb* mRNA levels were higher in white adipose tissue of the KLB AdipoTG mice but not at levels significantly greater than the chow fed WT levels indicating the ability to maintain *Klb* mRNA in white adipose tissue (Figure 2C). Consistent with *Klb* mRNA levels, KLB protein levels were reduced in white adipose tissue of both diet-induced obese WT and KLB AdipoTG mice compared to chow fed controls, but KLB protein levels in white adipose tissue of HFD-fed KLB AdipoTG mice were comparable to those of WT chow-fed mice (Supplementary Figure 1). Importantly, no increases in *Klb* mRNA levels were detected in the liver of KLB AdipoTG mice (Figure 2I), and *TdTomato* mRNA levels were undetectable in the liver (Figure 2J), thereby demonstrating specificity. As in our initial profiling (Figure 1C,E,G) and contrary to previous studies [17], [18], we did not detect any significant changes in *Fgfr1c* mRNA levels between WT and KLB AdipoTG mice (Supplementary Figure 2) under either chow of HFD fed conditions.

### 3.3 Maintenance of adipose β-klotho does not prevent the development of FGF21 resistance

**Figure 3:**
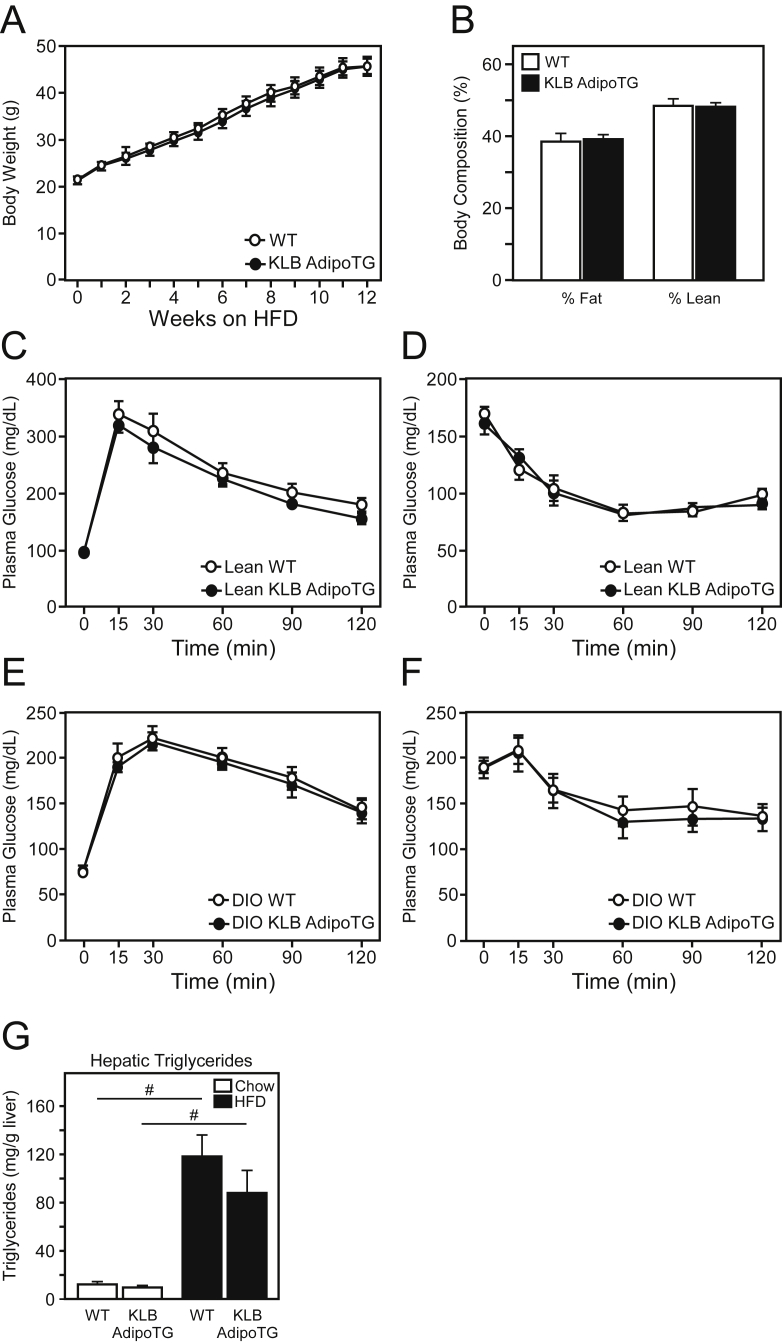
**Maintenance of β-klotho expression in white adipose tissue does not improve metabolic profiles**. (A) Body weight curves and (B) body composition of male WT and KLB AdipoTG mice on HFD for 12 weeks (n = 8–10/group). (C) Glucose tolerance (GTTs) and (D) insulin tolerance tests (ITTs) of lean, male WT and KLB AdipoTG mice (n = 6/group). (E) GTTs and (F) ITTs of diet-induced obese (DIO), male WT and KLB AdipoTG mice on HFD for 12 and 14 weeks, respectively (n = 6/group). (G) Hepatic triglyceride levels in DIO WT and KLB AdipoTG mice. Values are mean +/− SEM. (*, *P* < 0.05; ***, *P* < 0.005; and #, P < 0.001 compared to WT).

To determine whether maintenance of KLB expression in adipose tissue improves FGF21 sensitivity and prevents FGF21 resistance, we assessed the phenotype of KLB AdipoTG mice on either chow or HFD. Notably, no significant changes in body weight or body composition were observed between WT and KLB AdipoTG mice on either a chow or HFD (Figure 3A,B). Contrary to our hypothesis, no significant difference in glucose homeostasis or insulin sensitivity was observed in lean or DIO KLB AdipoTG mice as assessed by glucose and insulin tolerance tests, respectively (Figure 3C–F). Since FGF21 is known to have pronounced effects on hepatic lipid metabolism, we next assessed hepatic triglyceride levels in DIO WT and KLB AdipoTG mice. Consistent with no change in glucose and insulin tolerance tests, no significant difference in hepatic triglycerides was observed between lean or DIO WT and KLB AdipoTG mice (Figure 3G). Moreover, no significant differences in hepatic gene expression (Supplementary Figure 2) or serum parameters were observed between genotypes, except for a slight increase in plasma triglycerides and reduction in plasma ketones in DIO KLB AdipoTG mice (Supplementary Table 1).

### 3.4 FGF21 signaling remains impaired in white adipose tissue of KLB AdipoTG mice

**Figure 4:**
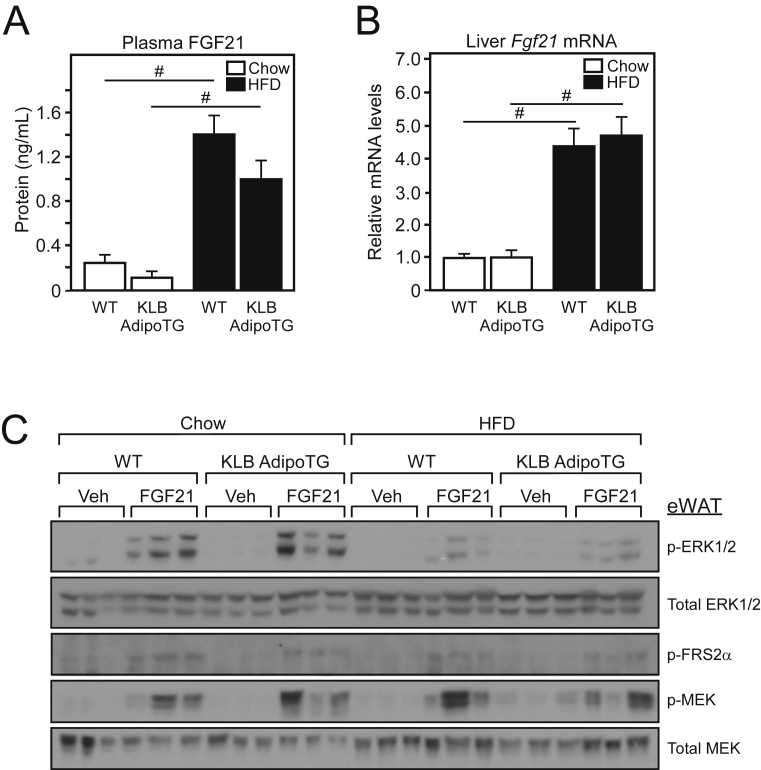
**White adipose tissue and systemic FGF21 sensitivity is not improved by preservation of β-klotho expression**. (A) Plasma FGF21 and (B) hepatic *Fgf21* mRNA levels in WT and KLB AdipoTG mice fed chow or HFD for 14 weeks (n = 8–9/group). (C) Western blot analysis of phospho-ERK1/2, total ERK1/2, phospho-FRS2α, phospho-MEK, and total MEK from eWAT of chow and HFD fed WT and KLB AdipoTG mice administered either vehicle or FGF21 (0.1 mg/kg) for 15 min. Values are mean +/− SEM. (#, P < 0.001 compared to WT).

Despite maintaining adipose β-klotho expression, plasma FGF21, and hepatic *Fgf21* mRNA levels were significantly elevated in DIO KLB AdipoTG mice compared to lean KLB AdipoTG mice (Figure 4A,B), suggesting no effect on sensitivity to endogenous FGF21. Therefore, we next assessed FGF21 signaling in eWAT of WT and KLB AdipoTG mice in response to low dose FGF21 administration. Under normal conditions, activation of the FGF21 receptor complex initiates a signaling cascade involving FRS2α and ERK1/2 signaling [23]. FGF21 administration to both lean and DIO WT and KLB AdipoTG mice revealed that FGF21-mediated induction of ERK1/2 phosphorylation is impaired in both DIO WT and KLB AdipoTG mice compared to lean control mice (Figure 4C). Notably, however, phosphorylation of upstream signaling components including FRS2α and MEK was not significantly different between lean and DIO WT and KLB AdipoTG in response to FGF21 (Figure 4C). These data indicate that maintenance of adipose β-klotho expression in vivo does not prevent the induction of plasma FGF21 levels, and that impairment of FGF21 signaling in white adipose tissue during obesity can occur downstream of β-klotho at the level of ERK phosphorylation.

## 4 Discussion

Although FGF21 has well-known metabolic effects, little is known about the mechanisms regulating FGF21 sensitivity. In this study we examined whether the marked reduction in β-klotho levels in white adipose tissue observed during obesity contributes to impaired FGF21 sensitivity in vivo. To accomplish this, we generated an adipose-specific transgenic mouse model which maintains β-klotho protein expression specifically in adipose tissue. In contrast to a previous report [43], we found that maintaining β-klotho levels in adipose tissue did not improve sensitivity to endogenous or exogenous FGF21. The discrepancy in our study and the study by Samms et al. is likely due to differences in animal models. Samms et al. utilized the aP2 promoter to overexpress β-klotho in adipose tissues [43]. However, the aP2 promoter drives expression in adipose and non-adipose tissues including the central nervous system [44], which is also an important target of FGF21 action [45]. In contrast, our studies utilized Adiponectin-cre mice, in which Cre-recombinase is expressed specifically in adipose tissues [46], crossed with inKLB transgenic mice to maintain β-klotho expression specifically in adipose tissues.

Consistent with previous studies that demonstrated impaired ERK1/2 phosphorylation in adipose tissue of diet-induced obese mice in response to FGF21 [17], [18], we also observed impaired ERK1/2 phosphorylation in white adipose tissue of diet-induced obese mice following acute administration of low-dose FGF21 treatment. Importantly, this decrease in ERK1/2 phosphorylation was still observed in KLB AdipoTG mice, demonstrating that maintenance of β-klotho levels does not reverse impaired FGF21 signaling in white adipose tissue. Interestingly, we observed decreased levels of ERK1/2, but not FRS2α or MEK1/2, phosphorylation in white adipose tissue of DIO WT and KLB AdipoTG mice, indicating that mechanisms downstream of KLB expression impair FGF21 signaling in vivo. One potential explanation for this effect on ERK1/2 phosphorylation could be an increase in dephosphorylation of ERK1/2 by a phosphatase. Dual specificity phosphatases (DUSPs) are a family of phosphatases that target tyrosine and serine/threonine residues and function to regulate fibroblast growth factor function [47], [48]. Unbiased gene expression analyses have revealed that multiple DUSPs are upregulated in white adipose tissue in response to FGF21 administration [49], and DUSPs function in a negative feedback loop to mediate control of intracellular ERK signaling [48]. Notably, one DUSP in particular, DUSP6, regulates FGF signaling [47] and is significantly increased in white adipose tissue during diet-induced obesity [50]. Further, DUSP6 knockout mice [50] exhibit phenotypic similarities to FGF21 transgenic mice [28] including increased insulin sensitivity and resistance to diet-induced weight gain. Thus, additional studies are necessary to examine the contribution of DUSP activity in regulating FGF21 sensitivity in adipose tissue.

In summary, we find that FGF21-stimultated ERK1/2 phosphorylation is impaired in white adipose tissue of diet-induced obese mice, and this impairment in white adipose FGF21 signaling is not prevented by maintaining β-klotho expression. While impaired FGF21-stimulated ERK1/2 phosphorylation in white adipose tissue has been proposed as evidence of FGF21 resistance during obesity, this impairment in FGF21 signaling could actually function as a beneficial physiological adaptation for excess nutrient disposal. Indeed, a recent publication demonstrated that acute FGF21 administration promotes lipid uptake in white adipose tissue under lean conditions, but redirects lipid catabolism to brown adipose tissue during obese conditions [37]. Consistent with these data, loss of circulating levels of FGF21 impairs glucose uptake in brown adipose tissue, but not white adipose tissues, during high fat diet feeding [14]. Although future studies are needed to test if white adipose tissue resistance to FGF21 is an adaptive mechanism, taken together with other studies, our data indicate that mechanisms other than β-klotho expression mediate the development of FGF21 resistance specifically in white adipose tissue under obesogenic conditions, and that this selective resistance may be a physiological adaptation to promote substrate utilization by other peripheral tissues.
