# Supplementary material for: FGF21 resistance is not mediated by downregulation of beta-klotho expression in white adipose tissue
Source: Mol Metab. 2017 Mar 27;6(6):602–10. doi: 10.1016/j.molmet.2017.03.009 (PMC5444074; doi:10.1016/j.molmet.2017.03.009)
Supplement: Supplementary file 1 [file mmc1.pdf]

SUPPLEMENTARY FIGURES AND TABLE

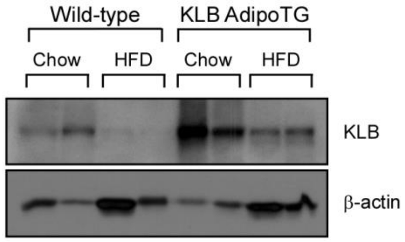

Supplementary Figure 1. Western blot analysis of KLB protein expression in epididymal white adipose tissue of wild-type (WT) and KLB adipose-specific transgenic mice (KLB AdipoTG) fed chow or HFD for 14 weeks.

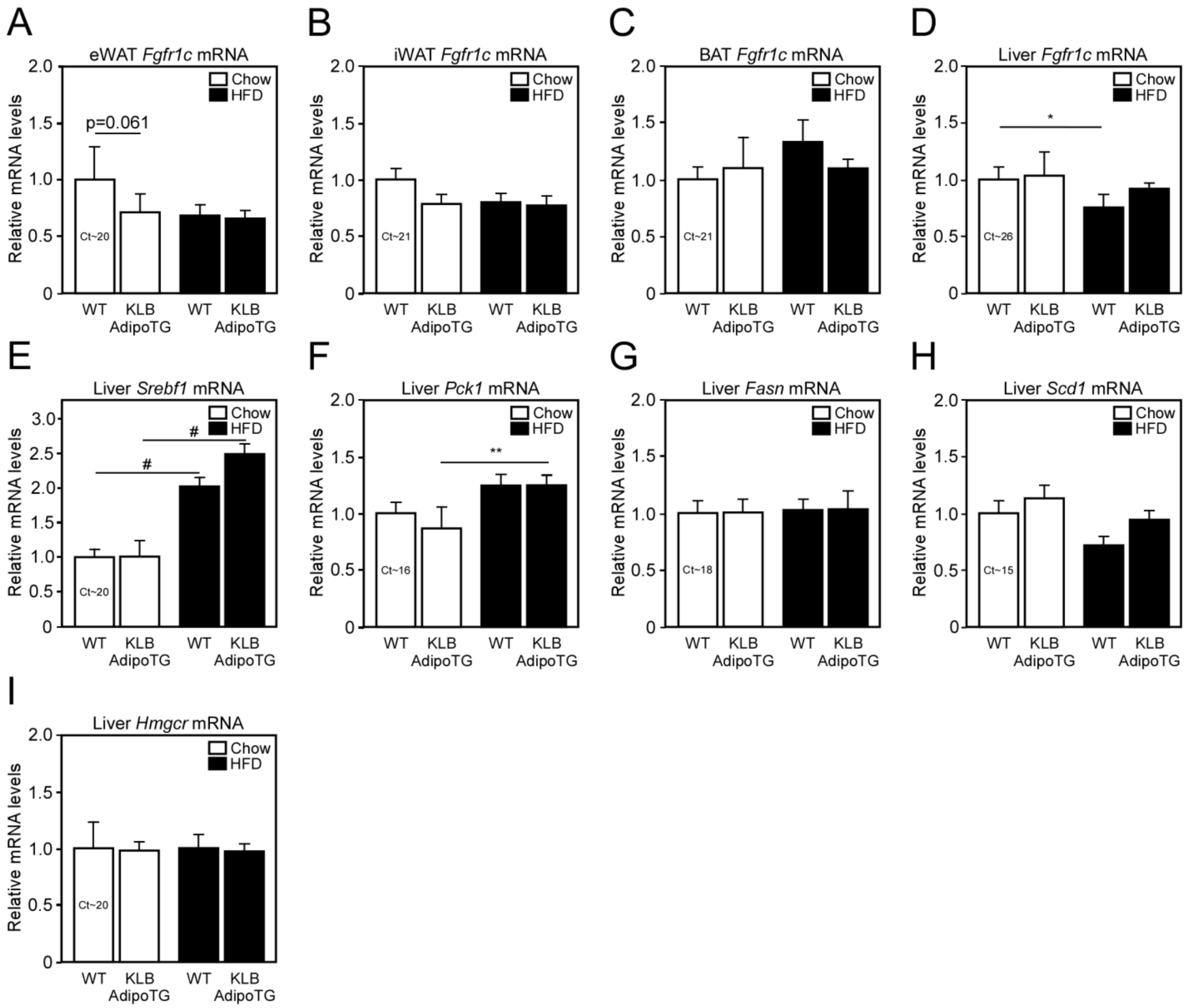

Supplementary Figure 2. Gene expression in wild-type (WT) and KLB adipose-specific transgenic mice (KLB AdipoTG) fed chow or HFD for 14 weeks as determined by QPCR. *Fgfr1c* mRNA expression in (A) epididymal white adipose tissue (eWAT), (B) inguinal white adipose tissue (iWAT), (C) interscapular brown adipose tissue, and (D) liver. Hepatic gene expression for (E) *Srebf1*, (F) *Pck1*, (G) *Fasn*, (H) *Scd1*, and (I) *Hmgcr*. Values are mean +/- SEM. (*, *P*< 0.05; **, *P*< 0.01; ***, *P*< 0.005; and #, *P*<0.001 compared to WT).

Supplementary Table 1 – Plasma parameters of KLB AdipoTG mice. Plasma parameters of WT and KLB AdipoTG littermates on chow and high fat diet for 14 weeks. Data are presented as mean ± SEM. (*, $P < 0.05$).

	Chow Diet		High Fat Diet	
	WT	KLB AdipoTG	WT	KLB AdipoTG
n	9	8	10	9
Food Intake (g/day)	3.20 ± 0.10	3.26 ± 0.11	2.89 ± 0.09	2.91 ± 0.05
Glucose (mg/dL)	171.27 ± 6.86	179.33 ± 8.84	215.93 ± 13.40	209.60 ± 10.82
Triglycerides (mg/dL)	102.14 ± 7.16	120.41 ± 14.43	94.05 ± 5.12	114.25 ± 6.83 *
NEFAs (mmol/L)	0.67 ± 0.04	0.66 ± 0.0	0.47 ± 0.04	0.52 ± 0.02
Cholesterol (mg/dL)	128.86 ± 4.59	129.24 ± 4.69	231.58 ± 11.50	224.90 ± 11.29
Total Ketones	64.11 ± 10.85	45.88 ± 4.19	79.10 ± 7.03	61.28 ± 3.86 *
